# Prefrontal-Temporal Pathway Mediates the Cross-Modal and Cognitive Reorganization in Sensorineural Hearing Loss With or Without Tinnitus: A Multimodal MRI Study

**DOI:** 10.3389/fnins.2019.00222

**Published:** 2019-03-12

**Authors:** Ying Luan, Congxiao Wang, Yun Jiao, Tianyu Tang, Jian Zhang, Gao-Jun Teng

**Affiliations:** Jiangsu Key Laboratory of Molecular and Functional Imaging, Department of Radiology, Zhongda Hospital, Medical School of Southeast University, Nanjing, China

**Keywords:** sensorineural hearing loss, dorsolateral prefrontal cortex, multimodal MRI, resting-state functional connectivity, tract-based spatial statistics, cross-modal reorganization, cognition, tinnitus

## Abstract

**Objective:** Hearing loss, one main risk factor of tinnitus and hyperacusis, is believed to involve significant central functional abnormalities. The recruitment of the auditory cortex in non-auditory sensory and higher-order cognitive processing has been demonstrated in the hearing-deprived brain. The dorsolateral prefrontal cortex (dlPFC), which has dense anatomical connections with the auditory pathway, is known to play a crucial role in multi-sensory integration, auditory regulation, and cognitive processing. This study aimed to verify the role of the dlPFC in the cross-modal reorganization and cognitive participation of the auditory cortex in long-term sensorineural hearing loss (SNHL) by combining functional and structural measurements.

**Methods:** Thirty five patients with long-term bilateral SNHL and 35 matched healthy controls underwent structural imaging, resting-state functional magnetic resonance imaging (rs-fMRI), diffusion tensor imaging (DTI), and neuropsychological assessments. Ten SNHL patients were with subjective tinnitus.

**Results:** No differences in gray matter volume, spontaneous neural activity, or diffusion characteristics in the dlPFC were found between the SNHL and control groups. The functional connectivity (FC) between the dlPFC and the auditory cortex and visual areas, such as the cuneus, fusiform, lingual cortex, and calcarine sulcus was increased in patients with SNHL. ANOVA and *post hoc* tests revealed similar FC alterations in the SNHL patients with and without tinnitus when compared with the normal hearing controls, and SNHL patients with and without tinnitus showed no difference in the dlPFC FC. The FC in the auditory cortex was associated with the symbol digit modality test (SDMT) scores in the SNHL patients, which reflect attentional function, processing speed, and visual working memory. Hearing-related FC with the dlPFC was found in the lingual cortex. A tract-based spatial statistics (TBSS) analysis revealed decreased fractional anisotropy (FA) values, mainly in the temporal inferior fronto-occipital fasciculus (IFOF), which showed remarkable negative correlations with the mean hearing thresholds in SNHL.

**Conclusion:** Higher functional coupling between the dlPFC and auditory and visual areas, accompanied by decreased FA along the IFOF connecting the frontal cortex and the occipito-temporal area, might mediate cross-modal plasticity via top-down regulation and facilitate the involvement of the auditory cortex in higher-order cognitive processing following long-term SNHL.

## Introduction

Approximately half a billion people are suffering from disabling hearing deprivation, accounting for 6–8% of the global population (Wilson et al., 2017). Hearing loss is associated with several common hearing disorders, such as tinnitus and hyperacusis (Eggermont and Roberts, 2004; Knipper et al., 2013). Since the most discussed pathology of tinnitus and hyperacusis is the elevated central auditory gain as a response to the loss of peripheral auditory input, hearing loss is one of the main risk factors to develop these hearing impairments (Knipper et al., 2013). The exploration of the neural abnormalities of hearing loss might be essential to develop a better understanding of the neural mechanisms underlying tinnitus, hyperacusis or other hearing disorders. Previous research indicates that widespread functional reorganization, within both the central auditory system and the non-auditory brain, is involved in hearing loss. The association between hearing loss and the raised risk of cognitive and emotional impairments has been widely accepted (Smith and Pichora-Fuller, 2015; Rutherford et al., 2018). No specific treatment has been proven effective for hearing loss. Hearing aids and cochlear implants are two major ways to partially restore the perception of environmental sounds and speech, but they are of limited value, particularly with regard to the cognitive decline, affective disorders, and social isolation that occur in cases of severe or profound hearing loss (Dawes et al., 2015; Rutherford et al., 2018).

Cross-modal plasticity is an intrinsic ability of the central multi-sensory brain, and refers to the recruitment of resources from the deprived modality into the intact sensory processing; it is known as a compensatory adaption of wide-ranging brain circuits (Glick and Sharma, 2017). Cross-modal reorganization has been well-documented in hearing loss. A functional near-infrared spectroscopy study revealed a stronger response in the auditory cortex elicited by visual motion stimulation in deaf patients compared to normal-hearing controls (Dewey and Hartley, 2015). The N1 visual evoked potential was reported in the temporal lobe in patients with hearing loss, although they had received cochlear implants (Buckley and Tobey, 2011). The auditory cortex in patients with early moderate hearing loss also shows robust activation to somatosensory stimuli (Cardon and Sharma, 2018). However, the exact principles of where and how the cross-modal organization happens following hearing loss remain poorly understood. Although it has been proposed that this reorganization might result from changes in local connectivity, supported by altered sensory maps, recent opinions emphasize the promising role of long-ranging connections in cross-modal reorganization. Besides, those studies on cross-modal activation overlooked the spontaneous functional architecture associated with hearing deprivation, as revealed by a recent study that showed cross-modal functional coupling patterns were different between resting state and sensory stimuli (Pelland et al., 2017).

Apart from low-level sensory processing, accumulating evidence has indicated that the deprived auditory cortex is also recruited for higher-order cognitive functions. Language stimuli-evoked activation in the superior temporal gyrus (STG) of deaf patients shows enhanced functional coupling with frontal areas, which correlates with the language learning function (Que et al., 2018). Activation in the auditory cortex was also elicited during the visual working memory task in patients with early hearing loss, suggesting that the functional modification related to hearing loss might not be explained by a bottom-up mechanism only (Ding et al., 2015). These results indicate that cross-modal reorganization might also rely on interactions with the fronto-parietal areas, which are thought to process higher-order cognitive functions.

The dorsolateral PFC (dlPFC) plays a crucial role in top-down regulation (O’Reilly, 2010), multi-sensory integration (Fuster et al., 2000), and cognitive function, including working memory (Wang et al., 2015), decision making (Philiastides et al., 2011), and response inhibition (Gläscher et al., 2012). Anatomically, the dlPFC receives multiple sensory afferents from the sensory cortices (Jones and Powell, 1970; Romanski et al., 1999), which support its multi-sensory integration, notably for sight and sound. The dlPFC also directly projects axons to the thalamic reticular nucleus (TRN), including the auditory portion, which is involved in negative regulation of auditory thalamo-cortical communication (Zikopoulos and Barbas, 2006). The dense connections between the dlPFC and the sensory areas provide a convenient circuit for cross-modal plasticity and top-down regulation (Morrone, 2010). Hence, we hypothesize that the dlPFC might mediate the recruitment of the auditory cortex in cross-modal plasticity and higher-order cognitive functions in hearing loss.

To verify our hypotheses, we evaluated the structural and functional properties of the dlPFC by combining multimodal magnetic resonance imaging (MRI) techniques. We aimed to determine whether the functional coupling pattern of the dlPFC changes following long-term sensorineural hearing loss (SNHL) with and without accompanying tinnitus, with a particular focus on the multiple sensory areas, and its relevance to hearing perception and higher-order cognitive performance. We further evaluated the properties of the white matter microstructure of the prefrontal-temporal pathway using a tract-based spatial statistics (TBSS) approach. We aimed to identify the dlPFC as a potential biomarker underlying the cross-modal reorganization and cognitive participation of the auditory cortex related to SNHL.

## Materials and Methods

### Participants

Thirty-five patients with long-term bilateral SNHL were recruited for this study (all right-handed, 21 men and 14 women, 31–69 years old). All of these SNHL patients reported consistent hearing loss over the duration from the onset age of 3–42 years. Hearing loss was caused due to a clinical history of ototoxic drug application in one case of the SNHL subjects, others had no clear etiology. Ten of these SNHL patients were accompanied by mild subjective tinnitus. Thirty-five age-, sex-, and education-matched normal-hearing controls were also included in this study (all right-handed, 17 men and 18 women, age range: 32–69 years). All participants were Chinese Han. The criteria for the inclusion of the patients with long-term bilateral SNHL were as follows: (1) age range: 20–70 years; (2) clinically diagnosed SNHL with a disease duration above 3 years; (3) post-lingual hearing loss; (4) bilateral hearing loss with mean hearing thresholds above 25 dB HL for both ears. The criteria of exclusion for both the SNHL and control groups were as follows: clinically diagnosed Meniere’s disease and acoustic neuroma, a clinical history of head injury, cancer, stroke or otologic surgery, poorly controlled diabetes or hypertension, seizures, Multiple Sclerosis, Parkinson’s disease, Alzheimer’s disease, depression, schizophrenia, and other neuropsychiatric diseases. This research was approved by the Ethics Committee of Affiliated Zhongda Hospital of Southeast University. A written informed consent was obtained from each of the participants before the experiment. All procedures were performed in accordance with the Declaration of Helsinki.

### Audiological Assessment

The hearing thresholds were measured at the frequency of 250, 500, 1000, 2000, 4000, and 8000 Hz via pure tone audiometry using a GSI-61 audiometer. The monaural mean hearing threshold was computed as the averaged value of the air conduction thresholds at 500, 1000, 2000, and 4000 Hz. The binaural mean hearing threshold was calculated by averaging the monaural mean hearing thresholds. Acoustic immittance was performed to exclude hearing loss due to conductive deafness. All subjects in the control group had mean thresholds <25 dB HL for both ears. All subjects in the SNHL group had mean thresholds >25 dB for both ears.

### Neuropsychological Assessment

A series of neuropsychological assessments that covered the relevant cognitive or emotional domains were conducted with all participants. The tests for each participant took approximately 1 h to complete and were administered by the same researcher in the same order. For cognitive assessment, the general cognitive state was evaluated by the Mini Mental State Examination (MMSE) (Galea and Woodward, 2005). The functions involving attention, visual scanning, and working memory, and the processing seed were assessed by the Symbol Digit Modalities Test (SDMT) (Smith, 1982). The episodic memory of verbal information was assessed by the Auditory Verbal Learning Test (AVLT) (Schmidt, 1996), comprising an immediate recall, a 5-min delayed recall, and a 20-min delayed recall test. For emotional assessment, possible depression, and anxiety states were determined via the Hamilton Depression Rating Scale (HAM-D) (Hamilton, 1960) and the Self-Rating Anxiety Scale (SAS) (Zung, 1971), respectively.

### MRI Data Acquisition

All participants underwent brain structural, resting state fMRI (rs-fMRI), and diffusion tensor imaging (DTI) scanning in a Siemens 3.0 T MRI scanner (Siemens, Erlangen, Germany) with a 12-channel head coil at the Department of Radiology, Affiliated Zhongda Hospital of Southeast University. Soft foam padding was used to alleviate head motion; earplugs and a headphone were used to alleviate the noise during scanning. The participants were instructed to keep their head still, eyes closed, and avoid thinking about anything in particular during the MRI scanning. Structural images were acquired using a high-resolution three-dimensional magnetization-prepared rapid gradient-echo (3D MPRAGE) T1-weighted sequence. The sequence parameters were as follows: repetition time: 1900 ms, echo time: 2.48 ms, flip angle: 9.0°, inversion time: 900 ms, slice number: 176, slice thickness: 1.0 mm, field of view: 250 mm × 250 mm, matrix: 256 × 256. Functional raw data were obtained using a gradient-recalled echo-planar imaging (GRE-EPI) sequence in an interleaved order. The sequence parameters were as follows: repetition time: 1900 ms, echo time: 2.48 ms, flip angle: 90.0°, slice number: 32, slice thickness: 4.0 mm, the field of view: 240 mm × 240 mm, matrix: 64 × 64, volumes number: 240. The DTI raw data were acquired using a single-shot echo planar imaging sequence (EPI) with the following parameters: repetition time: 5800 ms, echo time: 82 ms, flip angle: 90°, slice number: 31, slice thickness: 4.0 mm, *b*-values: 0 and 1000 s/mm^2^, acquired resolution: 2 mm × 2 mm × 2 mm, direction: 30, matrix: 128 × 128.

### Structural Data Analyses

To avoid the impact of structural damage on the functional measurements, we computed the volume of gray matter (GM) and white matter (WM) for each participant using the voxel-based morphometry (VBM) method via the SPM8 toolbox. The T1-weighted images of each subject were segmented into the GM, WM, and cerebro-spinal fluid and then non-linearly normalized to the standard Montreal Neurological Institute (MNI) space. The images after normalization were smoothed with an 8-mm full-width at half-maximum (FWHM) Gaussian kernel. The whole-brain GM and WM volumes were calculated by estimating the segments. The voxel-based between-group difference in GM and WM was determined via analysis of variance (ANOVA) using a general linear model (GLM) with age, sex, and education included as nuisance covariates of no interest. Significant differences were determined to have a *p*-value threshold <0.05 corrected by the false discovery rate (FDR) method for multiple comparison correction. The region of interest (ROI)-based VBM analysis was used to compare the regional GM volume between two groups. The ROIs were defined from the Brodmann template as the bilateral dlPFC. The subject-specific GM data within the ROI were exacted from the normalized and smoothed GM images.

### Functional Data Preprocessing and Analyses

The Statistical Parametric Mapping software (SPM8^1^) and the Data Processing and Analysis for Brain Imaging V2.3 (DPABI^2^) toolbox were used to preprocess the fMRI data including the following steps: exclusion of the first 10 volumes; slice-timing for correcting the differences in acquisition time between slices; realignment for head motion correction; spatial normalization to a standard template in the MNI space using a DARTAL approach followed by reslicing into 3 mm × 3 mm × 3 mm voxel size; spatial smoothing with a Gaussian kernel size of 6 mm × 6 mm × 6 mm FWHM; linear detrending; nuisance covariate regression for the white matter and cerebrospinal fluid signal and the head motion parameters; bandpass filter with the frequency window from 0.01 to 0.1 Hz. Subjects were excluded if their head motion exceeded 2.0 mm in the x, y, z plane, or 2.0° of axial rotation. No participant was excluded in the current study due to head motion.

#### Functional Connectivity and Hearing-Related Functional Connectivity Analyses

The seed-based functional connectivity (FC) analysis was implemented using the Resting-state fMRI Data Analysis Toolkit (REST^3^). The region of interest (ROI) in the left and right dlPFC was defined using the Wake Forest University (WFU) PickAtlas software^4^ (Macaluso and Driver, 2001) from the Brodmann template. The mean time series for each ROI were extracted and the Pearson’s correlation coefficients were calculated between the mean time series of the ROIs and each voxel across the brain. Fisher’s r-to-z transformation was implemented for each voxel to improve normality. The FC *z*-score map was generated for each participant. The between-group differences between the SNHL and control groups were determined by the ANOVA analysis using a GLM with age, sex, and education as the nuisance covariates of no interest. Differences with a *p* < 0.05 were deemed statistically significant, using an FDR method for multiple comparison correction. A one-way ANOVA was performed to determine the main effect of group on dlPFC FC among SNHL without tinnitus, SNHL with tinnitus and control groups, with age, sex, and education as the nuisance covariates of no interest. Then the two-sample *t*-tests masked by the ANOVA main effect results were used to identify (1) SNHL patients without tinnitus vs. controls, (2) SNHL patients with tinnitus vs. healthy controls, and (3) SNHL patients without tinnitus vs. SNHL patients with tinnitus. Differences with a *p* < 0.05/3 were deemed statistically significant for *post hoc* two-sample *t*-tests, using an FDR method for multiple comparison correction. A hearing-related FC map was calculated by correlating the binaural mean hearing thresholds with the FC *z*-scores of each voxel, with the ROI set at the left and right dlPFC, respectively, in all SNHL patients, with age, sex, and education as the covariates. Significant differences were considered with a cluster threshold of *p* < 0.05 using the Gaussian Random Field method at a voxel height of *p* < 0.001.

#### Amplitude of Low-Frequency Fluctuation Calculation

The whole brain amplitude of low-frequency fluctuation (ALFF) was calculated for each participant using the REST software. The preprocessed time series of each voxel was transformed to a power spectrum via a fast-Fourier transform. The square root of the power spectrum was calculated at each frequency and averaged across a low-frequency (0.01–0.1 Hz) domain to acquire the amplitude (Zou et al., 2015). Then the individual ALFF map was transferred into a *z*-score map by subtracting the averaged ALFF value. The mean ALFF *z*-scores were extracted from the bilateral dlPFC for each participant.

### Diffusion Imaging Preprocessing and Analyses

The DTI data processing was performed using the FMRIB Software Library (FSL^5^). The skulls were removed using the brain extraction tool of FSL. FMRIB’s Diffusion Toolbox was used to correct head motion and the distortions resulting from the eddy currents, estimate the diffusion tensor, and generate the fractional anisotropy (FA) map and mean diffusion (MD) maps for each participant. Finally, the individual FA and MD maps were directly registered to the high-resolution FMRIB58 template in a 1 mm × 1 mm × 1 mm standard MNI152 space using a non-linear algorithm.

#### Diffusion Measurements of the dlPFC

The normalized FA and MD maps were spatially smoothed with a Gaussian kernel size of 6 mm × 6 mm × 6 mm FWHM. Again, the averaged FA and MD values of the bilateral dlPFC were obtained from each of the participants referring to the Brodmann template.

#### Tract-Based Spatial Statistics

The TBSS analysis was employed following the pipeline of FSL. Briefly, the mean WM tract skeleton was generated in the normalized space with the threshold of FA >0.2, representing the central fiber bundles across all participants. Then all FA maps were projected to the mean FA skeleton. The voxelwise FA values in the WM skeleton were compared between the SNHL group and the control group using a randomization test with 5000 permutations in FSL, with age, sex, and education included as covariates. The statistical significance was set at *p* < 0.05 and we corrected for multiple comparisons using the threshold-free cluster enhancement method (TFCE).

### Statistical Analyses

Group differences in age, education, mean hearing thresholds, results of neuropsychological tests, GM volume, ALFF, FA values and MD values in ROIs between SNHL patients and healthy controls were determined with a Student’s *t*-test. Group differences in age, education, and mean hearing thresholds among the SNHL with tinnitus, SNHL without tinnitus and healthy control group were determined with one-way ANOVA. Group difference in sex was determined by the chi-square test (SPSS, 19 software, Chicago, IL, United States). Differences with *p* < 0.05 were considered to be statistically significant. The correlations between the functional or structural parameters and clinical variants in the SNHL group were assessed using a partial correlation analysis, controlling for age, sex, and education. Differences with *p* < 0.05 were considered to be statistically significant. To determine the power of the differences observed from the seed-based FC analysis, we used the receiver operating characteristic (ROC) curve analysis and calculated the area under the curve. All data are presented as the mean ± standard deviation (SD).

## Results

### Demographic, Hearing, and Neuropsychological Characteristics

**FIGURE 1:**
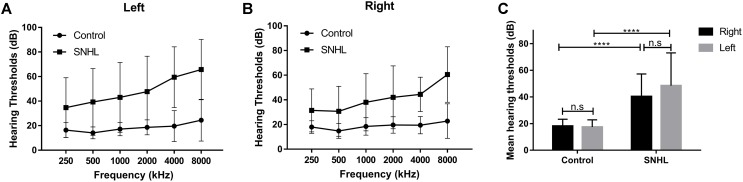
The hearing thresholds of **(A)** right side and **(B)** left side at 250, 500, 1000, 2000, 4000, and 8000 Hz in the control and SNHL groups. **(C)** No side differences in mean hearing thresholds are shown for either group. The mean hearing thresholds for each side in patients with SNHL are significantly higher than in healthy controls. The data are shown as mean ± standard deviation. ^∗∗∗∗^*p* < 0.0001.

Thirty five patients with SNHL and 35 healthy controls were included. All of the SNHL subjects had a history of long-term consistent hearing loss when this study was conducted. The mean duration of auditory deprivation in the SNHL subjects was 9.29 ± 8.95 years. Most of them acquired the hearing loss with no clear cause. There was no difference in age, sex, education, handedness, or ethnicity between the SNHL group and control group. The mean hearing thresholds of the left and right side were above 40 dB HL in the SNHL group (Figure 1). There was no significant difference between the two sides (*p* = 0.109). The mean hearing thresholds of both sides in the control group were significantly lower than in the SNHL group and were within the normal range (<20 dB HL). There was no significant difference between the two sides (*p* = 0.622). Relative to healthy controls, patients with SNHL presented significantly increased SAS and HAM-D scores, and significantly decreased AVLT-5 scores. No other between-group difference was found with regard to the MMSE, AVLT, AVLT-20, or SDMT tests. The detailed demographic, hearing, and neuropsychological results are shown in Table 1.

**Table 1:** Demographic, clinical, and neuropsychological characteristics of the patients with SNHL and healthy controls.

	SNHL patients (*n* = 35)	Healthy controls (*n* = 35)	t/χ^2^	*P*-value
Age (years)	54.49 ± 9.45	55.97 ± 7.80	-0.717	0.476
Gender (M/F)	21/14	17/18	0.921	0.337
Education (years)	11.09 ± 3.28	11.51 ± 3.46	-0.531	0.597
Handedness (R/L)	35/0	35/0		
Mean PTA of right ear (dB HL)	40.28 ± 16.98	18.12 ± 5.20	7.319	<0.0001^∗∗∗∗^
Mean PTA of left ear (dB HL)	48.48 ± 24.59	17.46 ± 5.44	7.253	<0.0001^∗∗∗∗^
Averaged PTA of both ears (dB HL)	44.38 ± 19.03	17.78 ± 4.89	7.954	<0.0001^∗∗∗∗^
Duration of hearing loss (years)	9.29 ± 8.95	–	–	–
**Neuropsychological tests**				
MMSE	29.49 ± 0.83	29.83 ± 0.47	-1.929	0.058
AVLT	17.09 ± 3.53	17.28 ± 5.43	-0.162	0.872
AVLT-5	6.11 ± 2.10	7.17 ± 1.54	-2.259	0.027^∗^
AVLT-20	6.06 ± 2.27	6.50 ± 2.05	-0.812	0.420
SDMT	37.66 ± 12.13	40.86 ± 8.24	-1.209	0.231
HAM-D	6.20 ± 3.90	4.21 ± 2.47	2.481	0.016^∗^
SAS	31.14 ± 7.98	27.41 ± 4.02	2.418	0.019^∗^


*All data are presented as mean ± standard deviation. ^∗^*p* < 0.05, ^∗∗∗∗^*p* < 0.0001. M/F, male/female; R/L, right/left; PTA, pure tone audiometry from 0.5 to 4 kHz; MMSE, Mini Mental State Examination; AVLT, Auditory Verbal Learning Test; AVLT-5, Auditory Verbal Learning Test 5-min recall; AVLT-20, Auditory Verbal Learning Test 20-min recall; SDMT, Symbol Digit Modalities Test; HAM-D, Hamilton Depression Rating Scale; SAS, Self-Rating Anxiety Scale.*

Among the 35 long-term SNHL patients, 10 subjects were accompanied by subjective tinnitus. As illustrated in Table 2, there was no significant difference among the SNHL patients with tinnitus, SNHL patients without tinnitus, and healthy controls in age (*F* = 0.835, *p* = 0.438), gender (χ^2^ = 1.485, *p* = 0.476) or education (*F* = 0.775, *p* = 0.465). *Post hoc* Least-Significance Difference (LSD) tests revealed no significant difference in the left mean hearing threshold (*p* = 0.263), right mean hearing threshold (*p* = 0.901), or bilateral mean hearing threshold (*p* = 0.440) between the SNHL patients with tinnitus and SNHL patients without tinnitus.

**Table 2:** Demographical and clinical comparisons of the SNHL subgroups and control group.

	SNHL patients without tinnitus (*n* = 25)	SNHL patients with tinnitus (*n* = 10)	Healthy controls (*n* = 35)	Significance, *p*-value
Age (years)	55.48 ± 9.79	52.00 ± 8.50	55.97 ± 7.80	*F* = 0.835, *p* = 0.438^a^
Gender (M/F)	16/9	5/5	17/18	χ^2^ = 1.485, *p* = 0.476
Education (years)	10.68 ± 3.19	12.10 ± 3.48	11.51 ± 3.46	*F* = 0.775, *p* = 0.465^a^
Handedness (R/L)	25/0	10/0	35/0	–
Mean PTA of right ear (dB HL)	40.46 ± 17.65	39.84 ± 16.05	18.12 ± 5.20	*p* = 0.901^b^
Mean PTA of left ear (dB HL)	50.72 ± 24.19	42.89 ± 25.99	17.46 ± 5.44	*p* = 0.263^b^
Averaged PTA of both ears (dB HL)	45.59 ± 18.62	41.37 ± 20.74	17.78 ± 4.89	*p* = 0.440^b^


*^a^ANOVA: SNHL without tinnitus, SNHL with tinnitus and healthy controls. χ^*2*^ test: SNHL without tinnitus, SNHL with tinnitus and healthy controls. ^*b*^*Post hoc* Least-Significance Difference (LSD) test: SNHL without tinnitus and SNHL with tinnitus*.

### Structural Results

No significant difference in the GM or WM volumes of the whole brain was observed between the SNHL and control groups at a threshold of *p* < 0.05 corrected by FDR.

### Regional Multimodal Measurements of the dlPFC

As illustrated by Table 3, no significant difference in the ALFF, GM volume, FA, or MD value was detected in the SNHL group when compared to the control group for both the left and right dlPFC.

**Table 3:** Multimodal measurements of the bilateral dlPFC.

Metrics	SNHL patients	Healthy controls	*t*	*P*-value
**Left dlPFC**				
ALFF *z*-score	-0.0178 ± 0.1323	0.0300 ± 0.2434	-1.023	0.310
GM volume	0.2978 ± 0.0363	0.2959 ± 0.0388	0.224	0.824
FA value	0.0699 ± 0.0028	0.0702 ± 0.0039	-0.412	0.682
MD value	0.0007 ± 0.0001	0.0007 ± 0.0001	1.709	0.092
**Right dlPFC**				
ALFF *z*-score	-0.0905 ± 0.1466	-0.0622 ± 0.2103	-0.653	0.516
GM volume	0.3225 ± 0.0425	0.3249 ± 0.0368	-0.243	0.809
FA value	0.0862 ± 0.0034	0.0875 ± 0.0046	-1.297	0.200
MD value	0.0007 ± 0.0001	0.0007 ± 0.0001	0.863	0.391


*Data are shown as mean ± standard deviation. dlPFC, dorsolateral prefrontal cortex; ALFF, amplitude of low-frequency fluctuation; GM, gray matter; FA, fractional anisotropy; MD, mean diffusion*.

### Seed-Based FC With Bilateral dlPFC

**FIGURE 2:**
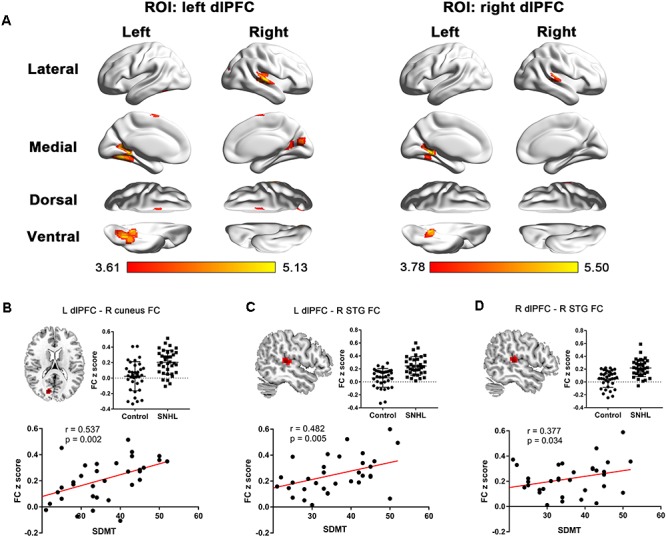
**(A)** The between-group difference distribution map of FC with the seed set to the left and right dlPFC independent of age, sex, or education (*p* < 0.05, corrected by FDR). Significantly increased FC is present in the red-yellow heat scales. The color bar below shows the *t*-scores obtained from the between-group comparisons. The SDMT scores are positively correlated with the left dlPFC-seeded FC in the right cuneus **(B)**, left dlPFC-seeded FC in the right STG **(C)**, and the right dlPFC-seeded FC in the right STG **(D)** in patients with SNHL, after controlling for age, sex, and education. FC, functional connectivity; dlPFC, dorsolateral prefrontal cortex; FDR, false discovery rate; SDMT, Symbol Digit Modalities Test; STG, superior temporal gyrus; ROI, region of interest; L, left; R, right.

**FIGURE 3:**
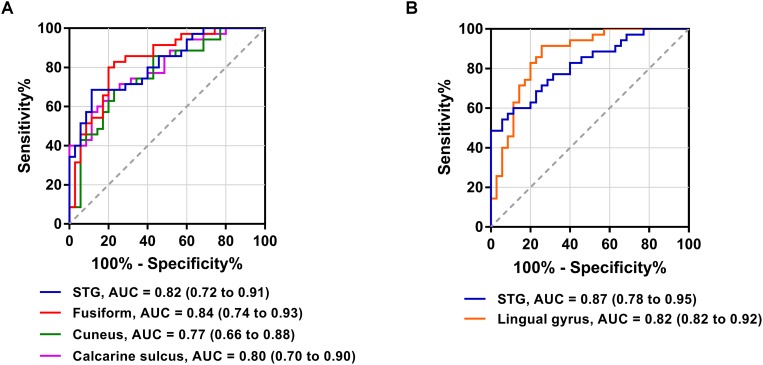
Receiver operating characteristic curves for differentiating patients with SNHL and healthy controls based on the significant FC with the left dlPFC **(A)** and right dlPFC **(B)**. AUC, area under the receiver operating characteristic curve.

The FC patterns of the left and right dlPFC were significantly different between the SNHL and control groups, as presented in Figure 2A and Table 4. When the ROI was set to the left dlPFC, the right superior temporal gyrus (STG), supplementary motor area (SMA), and several visual areas, including the right cuneus, calcarine sulcus, and left fusiform gyrus, showed significantly enhanced FC in the SNHL group. When the ROI was set to the right dlPFC, the right STG and left lingual gyrus showed significantly enhanced FC in the SNHL group. In the SNHL group, the SDMT scores were positively correlated with left dlPFC-seeded FC in the right cuneus and STG, and with right dlPFC-seeded FC in the right STG (Figure 2B–D), after controlling for the effects of age, sex, and education. The area under the receiver operating characteristic curve (AUC) of the temporal and occipital clusters with significant between-group differences in the FC with the left (Figure 3A) and right (Figure 3B) dlPFC were all above 0.75.

**Table 4:** Differences in FC in patients with SNHL compared with healthy controls when the ROI was set at the left and right dlPFC.

Brain region	BA	Voxel size	Peak MNI coordinates (mm)			Peak *t*-values
			*X*	*Y*	*Z*	
**Left dlPFC**						
**SNHL patients > healthy controls**						
Right cuneus	18	28	15	-81	18	4.3364
Right calcarine sulcus	17	25	27	-51	6	4.4442
Right superior temporal gyrus	22/41/42	87	51	-30	6	5.0824
Supplementary motor area	6	42	6	0	75	4.4006
Left fusiform gyrus	37	118	-21	-45	-3	5.1345
**Right dlPFC**						
**SNHL patients > Healthy controls**						
Right superior temporal gyrus	41/42	40	51	-27	6	5.3037
Left lingual gyrus	18	36	-21	-45	-3	5.5005


*FC, functional connectivity; ROI, region of interest; BA, Brodmann’s area; MNI, Montreal Neurological Institute*.

**FIGURE 4:**
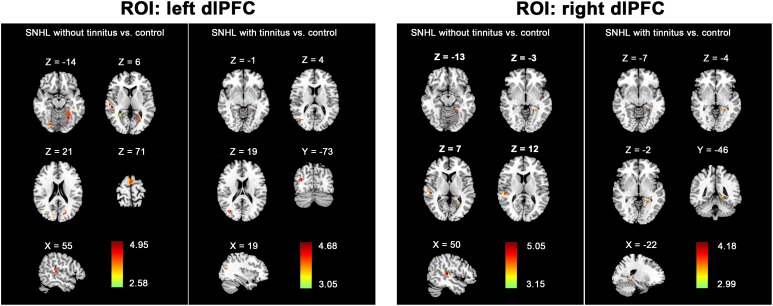
The between-group difference distribution map of FC with the seed set to the left and right dlPFC among the SNHL without tinnitus, SNHL with tinnitus, and healthy controls, independent of age, sex, or education (*p* < 0.05/3, corrected by FDR). Significantly increased FC is present in the red-yellow heat scales. The color bars show the t scores obtained from the between-group comparisons.

To further determine the effect of tinnitus associated with hearing loss on the dlPFC FC changes, we divided the SNHL patients into two subgroups and compared the dlPFC among the SNHL patients without tinnitus, SNHL patients with tinnitus and healthy controls (Figure 4). When the ROI was set to the left dlPFC, right STG, SMA, and several visual areas, including right fusiform, calcarine, and cuneus, and left lingual gyrus, fusiform gyrus, and calcarine sulcus, showed significantly enhanced FC when comparing the SNHL patients without tinnitus with healthy controls. Left lingual gyrus and fusiform gyrus showed significantly enhanced FC when comparing the SNHL patients with tinnitus with healthy controls. No significant difference was observed when comparing the SNHL patients without tinnitus with SNHL patients with tinnitus. When the ROI was set to the right dlPFC, the right STG and left lingual gyrus showed significantly increased FC when comparing the SNHL patients without tinnitus with healthy controls. Left lingual gyrus showed significantly increased FC when comparing the SNHL patients with tinnitus with healthy controls. No significant difference was observed when comparing the SNHL patients without tinnitus with SNHL patients with tinnitus.

### Hearing-Related dlPFC-Seeded FC

**FIGURE 5:**
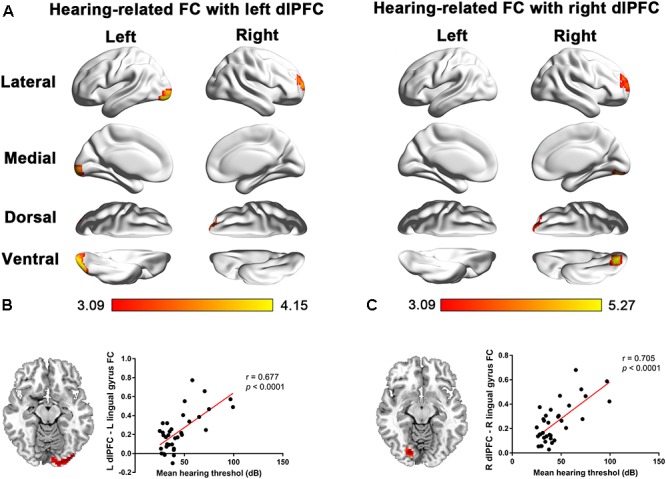
**(A)** Voxel-wise hearing-related FC patterns with the left and right dlPFC in patients with SNHL, independent of age, sex, and education. Significant positive correlations are presented in the red-yellow heat scales. The color bar below shows the *z*-scores. The scatterplots show the correlations in the patients with SNHL between the binaural mean hearing thresholds with the mean left dlPFC-seeded FC in the left lingual gyrus **(B)** and the mean right dlPFC-seeded FC in the right lingual gyrus **(C)**, which were shown to be significant by the voxel-wise correlation analysis, after controlling for the effects of age, sex, and education.

In patients with long-term SNHL, several brain regions showed hearing-related FC with the dlPFC. Table 5 presents the cluster size, cluster coordinates, and statistics. Figure 5A shows the anatomical locations of the significant clusters. FC in the left lingual gyrus and right anterior prefrontal cortex (aPFC) with the left dlPFC exhibited significant positive correlations with the binaural mean hearing thresholds with age, sex, and education considered as nuisance covariates. The mean FC *z*-scores in the left lingual gyrus were extracted and were found to be positively associated with the binaural mean hearing thresholds (*p* < 0.0001, Figure 5B), controlling for the effects of age, sex, and education.

**Table 5:** Hearing-related FC with the dlPFC in patients with SNHL.

Brain region	BA	Voxel size	Peak MNI coordinates (mm)			Peak *z*-values
			*X*	*Y*	*Z*	
**Left dlPFC**						
Left lingual gyrus	18	109	-18	-96	-3	4.1467
Right anterior prefrontal cortex	10	33	24	63	9	3.7206
**Right dlPFC**						
Right lingual gyrus	18	44	21	-81	-12	5.2721
Right anterior prefrontal cortex	10	74	27	57	24	3.9782


Similarly, the FC in the right lingual gyrus and right aPFC with the right dlPFC exhibited significant positive correlations with the binaural mean hearing thresholds, with age, sex, and education considered as nuisance covariates in patients with SNHL. The mean FC *z*-scores in the right lingual gyrus were extracted and positively associated with the binaural mean hearing thresholds (*p* < 0.0001, Figure 5C), controlling for the effects of age, sex, and education.

### Comparison of FA Values Between Patients With SNHL and Controls Using TBSS

**FIGURE 6:**
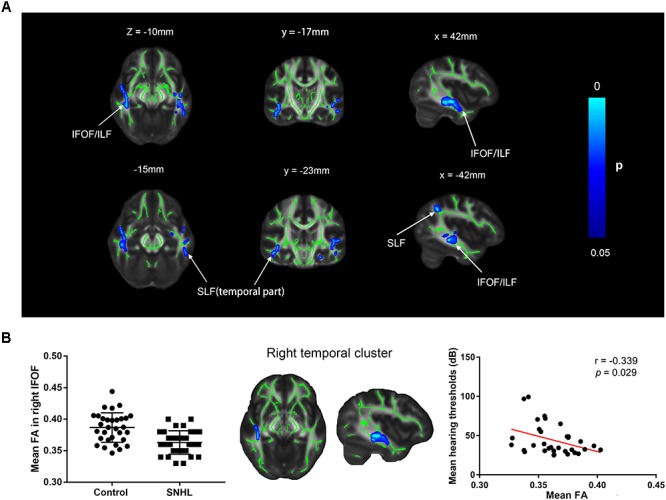
**(A)** Distribution map of the FA value that was significantly lower in the SNHL group compared with the control group based on the TBSS analysis. The mean FA skeleton (green) is overlaid with the blue clusters denoting significantly decreased FA values in patients with SNHL. The color bar on the left presents the *p*-value. The mean FA values in the significant cluster of the right temporal lobe are positively correlated with the binaural mean hearing thresholds in patients with SNHL, after controlling for the effects of the age, sex, and education **(B)**. FA, fractional anisotropy; IFOF, inferior fronto-occipital fasciculus; ILF, inferior longitudinal fasciculus; SLF, superior longitudinal fasciculus.

Several clusters with altered FA values were found in patients with SNHL compared with healthy controls (Figure 6A). The patients with SNHL showed reduced FA values within the WM skeleton in the bilateral inferior fronto-occipital fasciculus (IFOF), inferior longitudinal fasciculus (ILF), and superior longitudinal fasciculus (SLF), particularly in the temporal part near the auditory cortex. The mean FA value extracted from the significant cluster in the right temporal lobe exhibited a negative correlation with binaural mean hearing thresholds in patients with SNHL (Figure 6B), controlling the effects of age, sex, and education.

## Discussion

Recruitment of the auditory cortex into cross-modal and higher-order cognitive processing has been reported following hearing deprivation. We hypothesized that the connectivity between the dlPFC and auditory cortex or other brain areas might play a role in this. We assessed both local properties and long-ranging connectivity properties of the dlPFC by combining functional and structural measurements. The results showed that no ALFF, GM volume, FA, or MD changes occurred in the dlPFC. However, the distant functional coupling between the dlPFC and auditory cortex and multiple visual areas were remarkably increased in patients with SNHL independent of tinnitus. The long-ranging FC between the dlPFC and auditory cortex is associated with cognitive performance linking attentional function, information processing speed, and visual working memory in the subjects with SNHL. Higher levels of hearing loss contribute to stronger dlPFC-visual cortex coupling. Decreased FA values within the mean WM skeleton were observed in several central fiber bundles in SNHL, particularly in the temporal lobe near auditory cortex, which is associated with the degree of hearing loss.

In line with our local measurement results, most previous functional and structural imaging studies did not report significant regional changes of spontaneous neural activity, GM volume, or WM microstructure properties in the dlPFC in hearing loss (Li et al., 2012; Hribar et al., 2014; Lin et al., 2014; Rachakonda et al., 2014; Yang et al., 2014; Wang et al., 2016). Therefore, we speculate that the way by which the dlPFC regulates functional reorganization following SNHL is more likely via long-ranging connectivity rather than local functional or structural alterations. However, one recent rs-fMRI study reported increased ALFF in the dlPFC in patients with severe congenital hearing loss (Xia et al., 2017). This discrepancy could be related to the differences in the patients that were focused on, since in the current study, we recruited patients who acquired SNHL late and with a relatively wide range of hearing loss.

Prior behavioral, neuroimaging, and electrophysiological studies have provided convincing evidence of cross-modal plasticity following sensory modality deprivation, such as hearing loss and blindness; however, there is still much debate about where and how this kind of functional reorganization happens (Xia et al., 2017). Cross-modal plasticity and multisensory integration are influenced by bottom-up cochlear hearing loss (Sharma, 2014, 2017) and top-down regulation from cortical areas (Levin et al., 2007). One of the most robust candidates is the prefrontal top-down attentional control. The human brain has a limited capacity for information processing and can make a selection from the considerable inputs from the multiple sensory environments for dominant sensory stimuli. The competition among these sensory signals relies upon a bottom-up processing by which the salience stimuli evokes a robust cortical response, and a top-down regulation by which neural activity is biased toward a specific pattern relevant to the current behavioral intention (Kastner et al., 1998; Moore and Zirnsak, 2017). Selective attention regulates normal sensory processing. In cases of sensory deprivation, cross-modal reorganization is also mediated by the attentional transition from the deprived modality to the retained modalities via top-down modulation from the prefrontal cortex (PFC) (Morrone, 2010). Hearing loss can contribute to auditory attention deficits (Nagarkar and Panda, 2007; Park et al., 2016). A previous study revealed a cascade-of-control model during attention-demanding tasks, in which the dlPFC performs the top-down attentional control prior to the attentional selection modulated by the dorsal anterior cingulate cortex (dACC) (Silton et al., 2010). The dlPFC is one of the most described top-down control PFC regions in earlier studies (MacDonald et al., 2000; Dosenbach et al., 2006, 2008). Besides, cross-modal plasticity is assumed to rely on both multimodal brain structure and the unimodal brain areas. For example, visuo-tactile stimuli could elicit activation in the visual cortex when the tactile stimuli was on the same side as the visual target. The visual cortex also exhibited higher effective connectivity from the multimodal inferior parietal area and somatosensory cortex in the spatially congruent bimodal tasks. These findings indicate that the inferior parietal area, which is involved in the spatial and attentional processing, integrates the bimodal information and mediates the recruitment of the visual cortex to the sensory stimuli in cross-modal plasticity of spatial attention (Macaluso et al., 2000). Similar to the inferior parietal region, the dlPFC is also a multimodal brain area. In particular, the dlPFC is rich in audio-visual associative cells, supporting the involvement of the dlPFC in audio-visual association and integration (Fuster et al., 2000). The lesion of the dlPFC leads to deficiencies in discriminating auditory and visual stimuli (Petrides, 1986). Based on this notion, we proposed that the hypersynchrony of the dlPFC with both the auditory and visual cortices might reflect the adaptive reorganization of the audio-visual integration under circumstances of hearing loss, which facilitates the participation of the auditory cortex in visual processing. Besides, our voxelwise correlation analyses revealed that the hearing-related dlPFC FC was also located in the visual cortex, indicating that the reinforced functional integration between the dlPFC and the visual cortex was owing to the loss of peripheral hearing. Taken together, these findings suggest that hearing loss leads to cross-modal reorganization between the auditory and visual modalities, which is probably mediated by the dlPFC.

It has been proposed that the long-ranging connectivity between the PFC and the multiple sensory areas might mediate the recruitment of the deprived sensory cortices into high-order cognitive functions. The dlPFC has dense anatomical connections with the auditory cortex. In normal hearing subjects, these kinds of connections regulate auditory processing through attention control (Knight et al., 1995). The dlPFC lesions contribute to reduced attention effects during an auditory selective attention task (Knight et al., 1981). However, when SNHL is present, the innervation from the high-order cognitive circuit, such as the prefrontal areas, might lead the auditory cortex to participate more in higher-order cognitive functions. Moreover, the connections between the auditory cortex and the prefrontal multimodal areas are greater than other sensory cortices, such as the visual cortex (Beer et al., 2011; Yeterian et al., 2012). The takeover by prefrontal high-order cognitive areas in the auditory cortex might thus be a major pattern of functional reorganization in SNHL (Bedny, 2017). Findings from several recent studies on hearing loss have supported this hypothesis. The STG exhibited robust activation during a visual working memory task in deaf individuals, and this predicted the speed and accuracy of their working memory performances. Moreover, deaf individuals had stronger effective connectivity from the prefrontal eye field to the STG, implying the effects of the PFC on the participation of the auditory cortex in working memory processing (Ding et al., 2015). Resting-state FC between the STG and dorsal anterior cingulate cortex also predicted spatial working memory performances in patients with hearing loss (Ding et al., 2016). Apart from working memory, the auditory cortex is also implicated in language processing in hearing loss. A word recognition task led to enhanced activity in the auditory cortex, dlPFC, and inferior frontal cortex in older adults with hearing loss (Kuchinsky et al., 2016). Greater activation was also observed in the auditory cortex which depended on the degree of hearing loss during a sign language task (Lambertz et al., 2005). Larger and stronger response in auditory cortex to both natural speech and unintelligible speech understanding tasks was correlated with their speech understanding performances in the hearing loss patients, even though they already had the cochlear implants which partially restored their hearing (Olds et al., 2016). A recently proposed theory of cognitive pluripotency in the sensory cortices might account for these findings. During development, the auditory cortex is pluripotent, with the capability of performing a variety of low-level auditory and high-level cognitive functions based on its intrinsic physiology, including the local and long-ranging connectivity among the auditory pathway, other sensory cortices, and prefrontal high-order cognitive areas. The functional specialization depends on the input during development, confined by experience and connectivity (Gao and Suga, 2000). Under normal conditions, the dominant function of auditory cortex is processing the information in the acoustic signal, which is transmitted along the hierarchical bottom-up auditory processing system (Irvine, 2007). Altered information transmission causes cortical specialization. The loss of auditory input leads the intrinsic long-ranging connectivity from the prefrontal areas to recruit the auditory cortex into a series of cognitive processes (Bedny, 2017). In order to assess whether the dlPFC is involved in high-order cognitive takeover in the auditory cortex in SNHL, we correlated the elevated dlPFC FC in the STG with the SDMT performances in patients with SNHL. The finding of their association was in line with the previous theory that the long-ranging connectivity linking the prefrontal areas and auditory cortex contributes to functional modification in the auditory deprived brain. Previous event-related fMRI studies revealed activation in the fronto-parietal network and visual areas during SDMT tests, including the dlPFC, cuneus, and lingual gyrus (Forn et al., 2009, 2013; Akbar et al., 2016; Dobryakova et al., 2016). The SDMT requires the visual tracking, scanning, and attention regulated by the visual areas, as well as the attentional control, memory coding, working memory, and inferential reasoning involving the dlPFC (Silva et al., 2018). In the current study, the enhanced FC between the dlPFC and cuneus was also associated with the SDMT performances in the patients with SNHL, suggesting that the stronger functional integration between the dlPFC and visual cortex also contributed to faster information processing speed in SNHL. We propose that this result might be due to the hearing loss caused by the reinforced attention shift from the auditory modality to the visual modality, which was implicated in SDMT processing.

Regarding the seed-based FC results, remarkably enhanced dlPFC FC was mainly located in the STG and the visual cortex, including the cuneus, lingual gyrus, calcarine sulcus, and the fusiform gyrus. All of these abnormalities of dlPFC FC presented fair to excellent distinguishing power (0.7 < AUC < 1) when differentiating patients with SNHL from healthy controls, indicating that the dlPFC might be a potential imaging marker to identify patients with SNHL with cross-modal and higher-order cognitive reorganization in the auditory cortex. In addition, after we divided the SNHL patients into two subgroups according to whether they were accompanied by tinnitus, these two subgroups showed similar FC changes (i.e., increased dlPFC FC in the auditory and visual areas) when compared with the healthy controls, and no between-group difference. These findings indicate that the accompanying tinnitus makes negligible impacts on the hearing loss-related functional reorganization between the dlPFC with auditory, and visual areas. Tinnitus is generally accepted to be associated with the maladaptive plasticity of the auditory deafferentation caused by auditory deprivation (De Ridder et al., 2014). Previous functional imaging studies revealed the hyperactivity in the auditory area and the coactivation of some other brain areas, such as dlPFC (Vanneste et al., 2010). Electrophysiologic study has indicated that the tinnitus might be associated with the dysfunction in the dlPFC-dependent top-down inhibitory auditry modulation (Norena et al., 1999). However, we did not find additional impacts made by the tinnitus on the dlPFC FC beyond the SNHL. One of the possible explanations might be that the effects of the relatively mild tinnitus did not overweigh the profound effects of long-term bilateral hearing loss on the dlPFC FC pattern. Another interpretation might be the very limited sample size of the SNHL patients accompanied by the tinnitus and the heterogeneity of the tinnitus characteristics.

It is also important to understand the neural mechanisms underlying such reorganization in the auditory cortex in SNHL and distinguish the structural aspects from the functional aspects. Although the pathological substrates of DTI damage are still not clear, developing quantitative evaluations of the DTI metrics will provide a powerful tool to understand the microstructural features of the WM in the central brain. FA, a DTI measurement of the directional diffusion of water within the WM tracts, depends on the anatomical characteristics of the axonal microstructure (Scholz et al., 2009). A reduced FA value was observed in the bilateral IFOF, ILF, and SLF, particularly within the temporal lobe near the STG, in the SNHL group. These results were consistent with prior DTI studies conducted on deaf individuals (Kim et al., 2009; Husain et al., 2011; Stevens et al., 2017). The IFOF connects the lateral frontal cortex with the occipital and temporal lobes, the ILF links the ipsilateral occipital and temporal lobes, and the SLF connects the fronto-parietal and fronto-temporal areas (Catani et al., 2002). The temporal cortex facilitates these main WM tracts to communicate with the fronto-parietal and occipital regions. The reduced FA value in the temporal WM near the auditory cortex is speculated to be associated with the disuse in the auditory cortex due to the loss of the peripheral auditory input; lower FA with greater magnitudes of hearing loss found in the current study might support this assumption. Since the FA value is known to reflect the fiber density, fiber amount, and the degree of myelination (Schmithorst et al., 2005), one interpretation for the neural basis of such plasticity might be that the loss of peripheral auditory input resulted in the disuse-driven WM microstructural alterations in the temporal lobe near auditory cortex, such as axonal loss and demyelination (Song et al., 2002; Kim et al., 2009). In addition to the WM integrity damages of the fiber tracts, the FA value could also be affected by the distribution patterns of the WM fibers. Hence, another possible explanation might be the compensatory mechanism, which means other fibers expanding into this region result in more disordered WM tracts (Husain et al., 2011). Such compensation might provide the neuroanatomical substrate for cross-modal reorganization and the cognitive recruitment of the auditory cortex (Shiell and Zatorre, 2016; Karns et al., 2017). Although the exact underlying mechanism of both resting-state FC and the FA changes is difficult to understand, one possible interpretation might be that the functional connectivity is largely constrained by the structural connectivity (Sporns et al., 2009; Supekar et al., 2009). A previous study has indicated the association between neural activity and myelination by addressing the cellular substrates (Demerens et al., 1996). Hence, the intrinsic WM connectivity between the frontal and temporal areas, as an infrastructure, might mediate the functional coupling changes.

Surprisingly, we found that the functional abnormality of the STG was particularly located in the right side, even though the patients recruited in the current study all had bilateral SNHL. This might be due to the tendency of their higher hearing loss to be on the left side. Another possible explanation might be related to the specification of hemisphere dominance. Phonetic and language processing is left hemisphere dominant in the primary auditory cortex, and pitch processing is right hemisphere dominant in the primary auditory cortex (Zatorre et al., 1992). Prior studies indicate the important role of the left STG in sign language in individuals with hearing loss (MacSweeney et al., 2002; Sakai et al., 2005). Our patients with SNHL mostly had mild to moderate hearing loss, and none of these patients use sign language. We suggested that the right STG would be the predominately influenced area in the functional reorganization related to the loss of auditory input. Consistent with our finding, a previous study also found regional homogeneity in the right STG, but not the left STG, was altered in individuals with acquired bilateral hearing loss (Li et al., 2013).

Several limitations of this study must be acknowledged. First, because of the cross-sectional nature of this study, we were unable to track the dynamic progression of the SNHL. Further longitudinal studies will address this question. Second, although the total pool of subjects in the SNHL and the control groups was more than 30, the sample size was not large enough to divide the patients into different subgroups according to different cognitive or emotional states or by the severity or frequency of characteristics of hearing loss. We were able to partially overcome this problem by performing correlation analyses. Third, the blood-oxygen-level dependent (BOLD) signal is a reflection of local cerebral blood changes and is not a direct measure of neural activity. Combining rs-MRI with other non-invasive techniques, such as electroencephalography or magnetoencephalography, might be helpful to better understand the brain-wise neurophysiological mechanisms underlying SNHL and the cognitive associated with SNHL. Finally, the neuropsychological tests applied in the current study were relatively rudimentary. Future research will address the neural mechanisms underlying the more specific higher-order functions related to SNHL.

In summary, the current study substantiated the role of the prefrontal area, particularly the dlPFC, in the recruitment of the auditory area into cross-sensory and higher-order processing through a top-down control in conditions of long-term bilateral SNHL, by providing combined functional and structural neuroimaging evidence. Our findings enhance the understanding of cortical pluripotency and the underlying mechanisms of cross-modal reorganization and cognitive participation in the deprived auditory cortex.

## Author Contributions

YL conducted the experiments, recruited the participants, analyzed the data, and wrote the manuscript. CW helped with the integration of the data. YJ and TT helped with the analyses of the data. JZ helped the data collection. G-JT directed this study, designed the research, and gave key advice.

## Conflict of Interest Statement

The authors declare that the research was conducted in the absence of any commercial or financial relationships that could be construed as a potential conflict of interest.

## Footnotes

1 http://www.fil.ion.ucl.ac.uk/spm/

2 http://rfmri.org/dpabi/

3 http://www.restfmri.net/forum/

4 http://www.ansir.wfubmc.edu

5 http://www.fmrib.ox.ac.uk/fsl
